# A Quantitative Electroencephalography Study on Cochlear Implant-Induced Cortical Changes in Single-Sided Deafness with Tinnitus

**DOI:** 10.3389/fnhum.2017.00210

**Published:** 2017-05-18

**Authors:** Jae-Jin Song, Kyungsoo Kim, Woongsang Sunwoo, Griet Mertens, Paul Van de Heyning, Dirk De Ridder, Sven Vanneste, Sang-Youp Lee, Kyung-Joon Park, Hongsoo Choi, Ji-Woong Choi

**Affiliations:** ^1^Department of Otorhinolaryngology-Head and Neck Surgery, Seoul National University Bundang Hospital, Seongnam, South Korea; ^2^Department of Information and Communication Engineering, Daegu Gyeongbuk Institute of Science and Technology, Daegu, South Korea; ^3^Department of Otorhinolaryngology and Head and Neck Surgery, University Hospital Antwerp, Edegem, Belgium; ^4^Department of Surgical Sciences, Section of Neurosurgery, Dunedin School of Medicine, University of Otago, Dunedin, New Zealand; ^5^Lab for Clinical and Integrative Neuroscience, School of Behavioral and Brain Sciences, The University of Texas at Dallas, Richardson, TX, USA; ^6^Department of Robotics Engineering, Daegu Gyeongbuk Institute of Science and Technology, Daegu, South Korea

**Keywords:** single side deafness, tinnitus, cochlear implantation, electroencephalography, dynamic peripheral reafferentation

## Abstract

The mechanism of tinnitus suppression after cochlear implantation (CI) in single-sided deafness (SSD) is not fully understood. In this regard, by comparing pre- and post-CI quantitative electroencephalography (qEEG), we explored cortical changes relevant to tinnitus improvement. In SSD patients who underwent CI, qEEG data were collected: (1) before CI, (2) 6 months post-operatively with CI-on, and (3) 30 min after CI-off and source-localized cortical activity/functional connectivity analyses were performed. Compared to the pre-operative baseline, the CI-on condition demonstrated significantly decreased activity in the right auditory- and orbitofrontal cortices (OFC) for the delta frequency band as well as decreased connectivity between the auditory cortex/posterior cingulate cortex for the delta/beta2 bands. Meanwhile, compared to the CI-off condition, the CI-on condition displayed decreased activity in the right auditory cortices/OFC for the delta band, and in bilateral auditory cortices, left inferior frontal cortex/OFC for the gamma band. However, qEEG analyses showed no significant differences between the CI-off and baseline conditions. CI induced overall decreased cortical activity and functional connectivity. However, judging from no differences between the CI-off and baseline conditions, CI-induced cortical activity and functional connectivity changes are not by cortical plastic changes, but by dynamic peripheral reafferentation.

## Introduction

Tinnitus, the conscious perception of sound in the absence of a corresponding external acoustic stimulus (Baguley et al., 2013), afflicts 10–15% of the adult population and interferes severely with the quality of life of 5–26% of the affected population (Heller, 2003; Krog et al., 2010). The development of tinnitus is frequently deemed to be a neuroplastic response to sensory deprivation (Eggermont and Roberts, 2004; Song et al., 2012). This assumption is supported by a transient perception of tinnitus after experimentally induced partial (Schaette et al., 2012) and complete (Del Bo et al., 2008) temporary auditory deprivation in normal subjects, and was further reinforced by lack of tinnitus in congenitally deaf animal models (Eggermont and Kral, 2016). Furthermore, analogous to phantom limb pain, the tinnitus spectrum corresponds to auditory deprived frequencies (Norena et al., 2002).

In patients with severe peripheral auditory deafferentation, reafferentation of the ascending auditory nervous system with cochlear implants (CI) may abate tinnitus. Indeed, CI improved tinnitus significantly in 66–100%of CI users with bilateral profound hearing loss (Ruckenstein et al., 2001). Also, improvement of tinnitus by CI was reported in patients with single-sided deafness (SSD) and ipsilesional debilitating tinnitus (Punte et al., 2011). In a recent meta-analysis, CI showed a statistically significant improvement in the severity of tinnitus (Blasco and Redleaf, 2014). In this regard, CI is a promising treatment option for patients with SSD and combined severe tinnitus.

However, the mechanism of tinnitus suppression after CI in patients with SSD is not fully understood. In previous literature, several mechanisms of CI-mediated tinnitus suppression have been suggested. Some researchers have claimed that acoustic masking provided by CI is the primary mechanism of tinnitus suppression, by distracting attention from tinnitus (Andersson et al., 2009; Kleinjung et al., 2009), while others have suggested that plastic changes in the central auditory system by prolonged CI stimulation (Giraud et al., 2001) and electrical stimulation resulting in contralateral residual inhibition (Souliere et al., 1992) are possible mechanisms of tinnitus suppression. These assumptions are, however, based on inferential reasoning rather than data-driven analysis.

From this perspective, a study to explore post-CI changes in patients with SSD with regard to ongoing cortical activity may be of help in further understanding the mechanism of tinnitus alterations in SSD subjects after CI. By comparing pre- and post-CI source-localized quantitative electroencephalography (qEEG) findings, we attempted to find CI-driven cortical activity changes that may have abated subjective tinnitus in patients with SSD. Additionally, by analyzing changes in functional connectivity, we sought to reveal changes in functional connections of remote brain areas that may be responsible for the improvement of tinnitus after CI.

## Materials and Methods

### Participants

Four patients (three men and one woman) with unilateral acquired SSD (pure tone threshold >90 dB at 0.5, 1, 2, and 4 kHz) and ipsilateral tinnitus underwent pre-operative EEG and subsequent CI with a Med-EL device (Med-EL, Innsbruck, Austria). All patients presented with left-sided SSD and the median duration of deafness was 4.5 years (range, 9 months to 5 years). All four patients’ etiology of SSD was idiopathic sudden sensorineural hearing loss. The detailed demographic characteristics of the patients are summarized in **Table 1**.

**Table 1:** Demographic characteristics of the included subjects.

Subject number	Age (years)/sex	Contralateral hearing threshold (average of 0.5, 1, 2, and 4 kHz) (dB HL)	Duration of single-sided deafness	Etiology	Psychoacoustic characteristics of tinnitus	Frequency matching (Hz)	Perceived tinnitus loudness (dB SL)	Side of the cochlear implant	Name of the implanted device
1	53/female	20	4 years	Sudden sensorineural hearing loss	Pure tone	6000	20	Left	MED-EL Sonata ti 100 FLEX Soft electrode
2	64/male	23	5 years	Sudden sensorineural hearing loss	Pure tone	3000	10	Left	MEDEL Sonata ti 100 FLEX Soft electrode
3	47/male	15	9 months	Sudden sensorineural hearing loss	Pure tone	8000	30	Left	MED-EL Sonata ti 100 FLEX 24 electrode
4	49/male	15	5 years	Sudden sensorineural hearing loss	Narrow band noise	6000	40	Left	MED-EL Pulsar ci 100 Standard electrode

*dB HL, dB hearing level; dB SL, dB sensation level*.

The criteria for CI in patients with SSD and tinnitus were: (1) a duration of SSD < 10 years, (2) tinnitus development after SSD onset, and (3) tinnitus loudness on a numeric rating scale (NRS) ≥ 6 of 10 for at least 6 months that was intractable to conventional therapies including medication, tinnitus retraining therapy, and non-invasive neuromodulation such as transcranial magnetic stimulation or transcranial direct current stimulation. The exclusion criteria were: (1) severe depression with a Beck Depression Index (Beck and Steer, 1984) score > 16, and (2) a presumed etiology of tinnitus other than SSD. With regard to tinnitus loudness and tinnitus-related distress, all patients were evaluated using a NRS loudness score and a tinnitus questionnaire (TQ) (Goebel and Hiller, 1994) score.

This study and all related documents were approved by the ethics committee of Antwerp University Hospital. All patients gave written informed consent before enrollment. The study procedures were carried out in accordance with the relevant guidelines and regulations.

### EEG Recording

Pre-operative and 6-month post-operative EEGs were performed in all four patients. Pre-operative EEGs were recorded during resting-state for 5 min, while post-operative EEGs were recorded for 5 min under the following two conditions: (1) CI switch-on with a music stimulus (classical music from a radio channel) to the CI ear presented directly to the external audio processor via an audio cable at the most comfortable loudness level for each patient (CI-on); and (2) CI switch-off with no sound stimulus (CI-off).

Electroencephalograms were measured using the WinEEG software version 2.84.44 (Mitsar, St. Petersburg, Russia) in a room shielded against sound and stray electric fields with patients sitting upright with their eyes closed to reduce resting-state skin conductance levels in overall frequency bands (Barry et al., 2007, 2009). The EEG was sampled with 19 electrodes in the standard 10–20 International placements referenced to linked ears. While recording, impedance was maintained below 5 kΩ at all electrodes. Data were recorded with a sampling rate of 1,024 Hz using a 0.15 Hz high-pass filter and a 200 Hz low-pass filter. After initial recording, the data were processed offline by resampling to 128 Hz and band-pass filtering at 2–44 Hz by employing a fast Fourier transform filter with application of a Hanning window, and then imported into the Eureka! Software (Sherlin and Congedo, 2005) for precise artifact rejection before source-localization. All artifacts in the recorded EEG stream were removed meticulously by manual inspection.

The vigilance of all participants was checked by monitoring EEG streams to prevent unwanted changes, caused by drowsiness, such as alpha rhythm slowing or the appearance of spindles (Moazami-Goudarzi et al., 2010); no enrolled participant exhibited drowsiness-related EEG changes.

### Artifact Removal by Band-Limited Independent Component Analysis

Localization of the cortical resting-state or auditory evoked potentials in CI users via qEEG is confounded by stimulus artifacts produced by the implanted device itself. In a previous article, we described a successful method of CI artifact removal from specific bands in the EEG streams of patients with CIs using band-limited independent component analysis [BL-ICA, for further information, please refer to Kim et al. (2015)]. BL-ICA successfully removes artifacts by applying a narrow band-pass filter, which limits the number of sources and enhances the signal to noise ratio, thus allowing CI artifacts to be clearly detected and separated from other brain sources. By applying BL-ICA, all post-operative EEG data measured while listening to music were cleaned.

### Source Localization Analysis

Low-resolution brain electromagnetic tomography (LORETA)-KEY software^1^, dedicated to functional localization of current densities based on certain electrophysiological and neuroanatomical constraints, (Pascual-Marqui, 2002) was utilized to localize the cortical sources that generated the scalp-recorded electrical activity in each of the following eight frequency bands: delta (2–3.5 Hz), theta (4–7.5 Hz), alpha 1 (8–10 Hz), alpha 2 (10–12 Hz), beta 1 (13–18 Hz), beta 2 (18.5–21 Hz), beta 3 (21.5–30 Hz), and gamma (30.5–44 Hz) (Song et al., 2013a,b, 2014, 2015a,b; Vanneste et al., 2013; Kim et al., 2015, 2016). This software implements the lead field of Fuchs et al. (2002) that was derived from standard electrode positions realigned to a standard Montreal Neurological Institute (MNI)-152 head in combination with a boundary element method derived from the same standard anatomy (Jurcak et al., 2007). The LORETA-KEY anatomical template divides the neocortical MNI-152 volume, including the hippocampus and anterior cingulate cortex, into 6,239 voxels with dimensions of 5 mm × 5 mm × 5 mm, based on the Daemon Atlas (Lancaster et al., 2000). Anatomical labeling of significant clusters was performed automatically by a toolbox implemented in LORETA-KEY. The locations of significant clusters were initially investigated using the Anatomy toolbox (Eickhoff et al., 2005), and were reconfirmed using the Talairach and Tournoux atlas (Talairach and Tornoux, 1988). Renders were generated using the BrainNet Viewer^2^ (Xia et al., 2013).

### Functional Connectivity

Using the pre- and post-operative qEEG data, the extent of phase synchronization and coherence between the time series corresponding to different regions of interest (ROIs) were calculated to analyze functional connectivity. To calculate functional connectivity, we employed the built-in connectivity toolbox of the LORETA-KEY. This toolbox defines measures of linear- and non-linear dependence (i.e., coherence and phase synchronization) between multivariate time series. In the current study, we have calculated lagged linear coherence that excludes non-lagged parts of coherence which comprises effects of volume conduction, and effects of non-recorded sources that simultaneously drive recorded sources (Milz et al., 2014). For lagged linear coherence connectivity analysis, a total of 28 ROIs defined by Brodmann areas (BA) were selected as possible nodes based on previous literature on tinnitus: bilateral primary and secondary auditory cortices (A1s and A2s) (Rolls, 2004; Kringelbach, 2005), bilateral parahippocampus (PHC) (Landgrebe et al., 2009), bilateral dorsal/pregenual/subgenual anterior cingulate cortices (dACC/pgACC/sgACC) (Vanneste et al., 2010; De Ridder et al., 2011), bilateral posterior cingulate cortices (PCC) (Vanneste et al., 2010; Schecklmann et al., 2011), bilateral insula, bilateral precuneus, and bilateral orbitofrontal cortices (OFC) (Vanneste et al., 2010; De Ridder et al., 2011).

### Statistical Analysis

To identify cortical activity differences between pre-operative resting-state and post-operative sound stimuli-induced cortical activity, between pre-operative resting-state and post-operative device-off state activity, and between post-operative device-on with sound stimuli and device-off state activity (“CI-on – CI-off”), voxel-by-voxel analysis using LORETA-KEY was performed for the eight frequency band between-condition comparisons of the current density distribution. Also, regression analyses were performed to compare between “CI-on – CI-off” and percent improvement in tinnitus loudness and between “CI-on – CI-off” and percent improvement in TQ score. For source-localized group comparison analyses, statistical non-parametric mapping (SnPM) of LORETA-KEY images was performed for each contrast using LORETA-KEY’s built-in voxelwise randomization tests (5000 permutations) and employing a log-*F*-ratio statistic for independent groups with a threshold of *P* < 0.01. A correction for multiple comparisons in SnPM using random permutations (5000 permutations in the current study) has been proven to give results similar to those obtained from a comparable Statistical Parametric Mapping approach using a general linear model with multiple comparison corrections derived from random field theory (Holmes et al., 1996; Nichols and Holmes, 2002). Additionally, power spectral density (PSD) was calculated by EEGLAB toolbox (Delorme and Makeig, 2004). Topography was described based on PSD in 19 channels. The red, green, and blue colors in the topography represent maximum, mean, and minimum power, respectively, in specific bands such as delta and gamma.

For lagged linear connectivity differences, we compared differences between the pre-operative baseline, post-operative CI-off, and post-operative CI-on with music stimuli conditions, employing the *t*-statistics for groups with a threshold of *P* < 0.05, and also corrected for multiple comparisons by performing LORETA-KEY-built-in voxelwise randomization tests (5000 permutations).

All other descriptive statistical analyses were performed using the SPSS software version 20.0 (SPSS Inc., Chicago, IL, USA). For all analyses, descriptive statistical significance was set at *P* < 0.05.

## Results

### Comparison of Changes in Visual Analog Scale Tinnitus Loudness and Tinnitus Questionnaire Scores in all Patients

**Table 2** summarizes the pre- and post-operative comparisons of NRS tinnitus loudness and TQ scores. All four patients showed improved NRS loudness and TQ scores under the post-operative CI-on state compared with the pre-operative baseline. Also, compared to pre-operative NRS loudness (median, 8.5; range, 7–9) and TQ scores (median, 61; range, 52–78), the post-operative CI-on state showed improved NRS loudness (median, 4; range, 3–6) and TQ scores (median, 42.5; range, 29–56) with a trend-level significance (*P* = 0.068; *Z* = 1.826 for both comparisons, Wilcoxon signed rank test). When we compared the post-operative CI-on and off states, the CI-on state showed tinnitus alleviation with regard to NRS loudness and TQ score compared with CI-off NRS loudness (median, 8.5; range, 6–9) and TQ score (median, 61; range, 52–76) with a trend-level significance (*P* = 0.068; *Z* = 1.826 for both comparisons, Wilcoxon signed rank test). However, the comparison between the pre-operative baseline and the post-operative CI-off state showed no differences with regard to NRS loudness and TQ score (*P* = 0.317; *Z* = 1.00 and *P* = 0.564; *Z* = 0.577, respectively, Wilcoxon signed rank test) (**Table 2**).

**Table 2:** Pre- and post-operative comparison of numeric rating scale tinnitus loudness and tinnitus questionnaire scores in all patients (the order of the subjects are the same as **Table 1**).

Subject number	Pre-operative NRS loudness	Pre-operative TQ score	Post-operative NRS loudness (CI-on with music stimuli)	Post-operative TQ score (CI-on with music stimuli)	Post-operative NRS loudness (CI-off)	Post-operative TQ score (CI-off)
1	8	78	3	41	8	76
2	7	60	5	44	6	58
3	9	52	3	29	9	52
4	9	62	6	56	9	64

*NRS, numeric rating scale; TQ, tinnitus questionnaire*.

Meanwhile, regression analyses comparing between “CI-on – CI-off” and percent improvement in tinnitus loudness and between “CI-on – CI-off” and percent improvement in TQ score did not reveal any significant correlations between cortical activity changes and percent improvement in tinnitus loudness or TQ score.

### Group Comparison with Regard to Source-Localized Activity and Functional Connectivity

#### Post-operative CI-On versus Pre-operative Baseline

**FIGURE 1:**
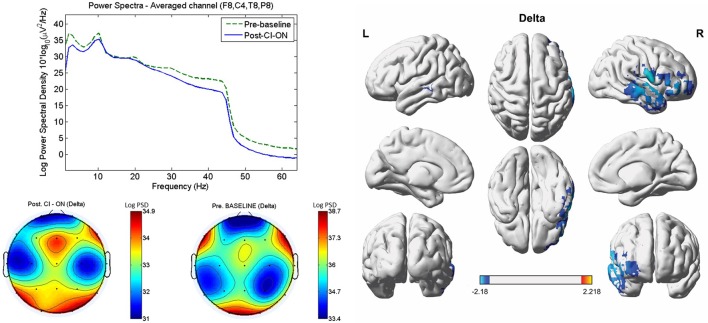
**Comparisons between the post-operative cochlear implant (CI)-on condition and the pre-operative baseline with regard to power spectra, sensor topography, and source-localized activity**. As compared with the pre-operative baseline, the post-operative CI-on condition resulted in significantly decreased activity in the right primary (BAs 41 and 42) and secondary (BAs 21 and 22) auditory cortices, and in the right orbitofrontal cortex (BAs 10 and 11) for the delta frequency band (*P* < 0.01). L, left; R, right. Color bar (right bottom) presents *t*-value. PSD, power spectral density.

Compared with the pre-operative baseline, the post-operative CI-on condition resulted in significantly decreased activity in the right A1 (BAs 41 and 42) and A2 (BAs 21 and 22) and in the right OFC (BAs 10 and 11) for the delta frequency band (*P* < 0.01) (**Figure 1**). For the other seven frequency bands, no significant differences with regard to source-localized activity were found between the two conditions.

**FIGURE 2:**
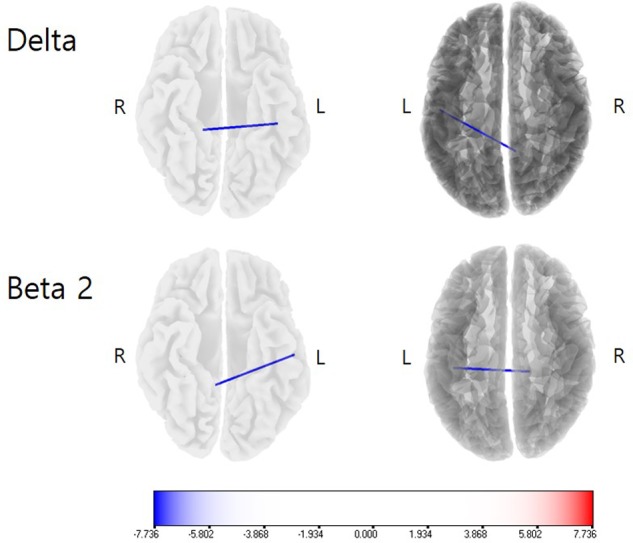
**Lagged linear connectivity comparisons between the post-operative cochlear implant (CI)-on condition and the pre-operative baseline**. The patients showed decreased functional connectivity between the PCC and A1 for the delta frequency band and between the PCC and A2 for the beta 2 band for the post-operative CI-on state, as compared with the pre-operative baseline. Color bar presents *t*-value.

On lagged linear connectivity comparison, the patients showed decreased functional connectivity between the PCC and A1 for the delta frequency band and between the PCC and A2 for the beta 2 band for the post-operative CI-on state compared with pre-operative baseline (**Figure 2**).

#### Post-Operative CI-On versus CI-Off

**FIGURE 3:**
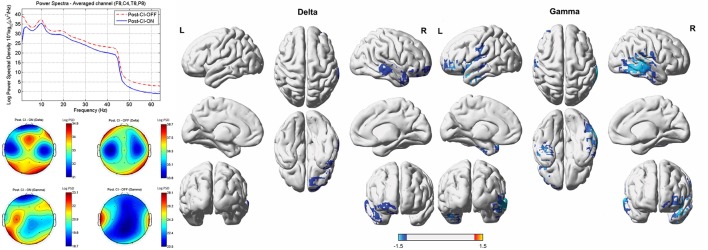
**Comparisons between the post-operative cochlear implant (CI)-on and off conditions with regard to power spectra, sensor topography, and source-localized activity**. Compared to the cortical activity of the CI-off condition, the subjects demonstrated significantly decreased activity in the right primary (BAs 41 and 42) and secondary (BAs 21 and 22) auditory cortices (A1 and A2), and right orbitofrontal cortex (OFC, BAs 10 and 11) for the delta frequency band and in the right A1 and A2, left A2, left temporopolar cortex (BA 38), left inferior frontal cortex (BA 47), and left OFC for the gamma band for the CI-on with music stimulus condition. L, left; R, right. Color bar (right bottom) presents *t*-value. PSD, power spectral density.

Compared to the cortical activity of the CI-off condition, the subjects demonstrated significantly decreased activity in the right A1 (BAs 41 and 42) and A2 (BAs 21 and 22) and right OFC (BAs 10 and 11) for the delta frequency band, and in the right A1 and A2, left A2, left temporopolar cortex (TPC, BA 38), left inferior frontal cortex (IFC, BA 47), and left OFC for the gamma band of the CI-on condition (*P* < 0.01) (**Figure 3**).

On lagged linear connectivity comparison, no significant differences were found between the two conditions for all eight frequency bands.

#### Post-operative CI-Off versus Pre-operative Baseline

Neither source-localized cortical activity comparisons nor lagged linear functional connectivity analysis showed statistically significant differences between the CI-off and pre-operative baseline conditions for all eight frequency bands.

## Discussion

In the current study, we investigated post-CI changes in patients with SSD with regard to source-localized cortical activity and functional connectivity. In short, the CI-on condition resulted in decreased cortical activity as compared with both the CI-off and pre-operative baseline conditions, but the CI-off and pre-operative baseline conditions showed no significant differences.

### Alleviation of Tinnitus and Tinnitus-Related Distress by Peripheral Reafferentation-Induced Cortical Deactivation

All four subjects in the current study showed improvements in NRS tinnitus loudness and TQ score. Although these improvements were only marginally significant both for NRS tinnitus loudness and TQ score (*P* = 0.068 for both parameters), considering the small number of included subjects, the improvements can be regarded to indicate a meaningful alleviation of tinnitus and tinnitus-related loudness.

On source-localized cortical activity analysis, as compared with the pre-operative baseline, the post-operative CI-on condition demonstrated significantly decreased activity in the right A1 and A2, and in the right OFC for the delta frequency band. Previous studies both in animals (Engineer et al., 2011) and in human subjects (van der Loo et al., 2009) have demonstrated that the auditory cortex (AC) plays an important role in tinnitus perception. A recent meta-analysis on positron emission tomography (PET) studies in tinnitus patients has also reported increased regional cerebral blood flow in the A1 and A2 (Song et al., 2012). Moreover, perceived tinnitus loudness is correlated with increased contralateral source-localized activity in the AC (van der Loo et al., 2009). In this regard, the significantly decreased activity in the A1 and A2 in patients with SSD after CI, compared with the pre-operative baseline, may be associated with the improvement of tinnitus loudness in these subjects.

Moreover, significantly decreased functional connectivity between the A1 and PCC for the delta frequency band and between the A2 and PCC for the beta 2 band under the CI-on condition, as compared to those under the pre-operative baseline, may also be related to the improvement of tinnitus loudness in these SSD subjects. PCC has been posited to be an important component of the brain’s default mode network (DMN) (Raichle et al., 2001; Raichle and Snyder, 2007), which is a set of cortical areas activated when a subject is occupied with internally focused tasks (Schlee et al., 2012). In persistent vegetative state patients, auditory stimulation-induced cortical activation is restricted to the A1, without functional connectivity to the areas comprising the DMN, including the PCC (Laureys et al., 2000; Boly et al., 2004). In other words, functional connectivity between the A1 and the PCC is crucial for conscious auditory perception. In a recent study that evaluated the correlation between pre-CI cortical activity and the extent of tinnitus improvement, increased activity of the PCC for the delta band and increased functional connectivity between the A1 and the PCC for the delta band were negatively correlated with the percent improvement of tinnitus loudness (Song et al., 2013b). This is in line with the current results showing the significantly decreased functional connectivity between the A1 and PCC for the delta frequency band under the CI-on condition as compared to those under the pre-operative baseline. That is, functional decoupling between the A1 and PCC for the delta band by CI may be associated with the improvement of tinnitus under the CI-on condition as compared with the pre-operative baseline condition. Also, the delta and beta 2 frequency bands has been found to be important in the integrity of the DMN in previous EEG studies (Neuner et al., 2014; Thatcher et al., 2014). Considering this, functional decoupling of the A1/A2 from a component of DMN for the delta and beta 2 frequency bands may have hindered conscious perception of the abnormal activity in the auditory cortices and thus associated with the improvement of tinnitus loudness. Thus, decreased functional connectivity between the A1/A2 and the PCC may be associated with the improvement of tinnitus loudness in these subjects.

Meanwhile, significantly decreased activity in the right OFC for the delta frequency band after CI compared with the pre-operative baseline may be associated with the improvement of the TQ score (i.e., tinnitus-related distress). The OFC has been suggested to be important for emotional processing of sounds (Blood et al., 1999; Vanneste and De Ridder, 2012) and also plays an important role in the top-down modulation of peripheral physiological responses to emotional experiences (Critchley et al., 2004). Additionally, the aforementioned correlation study between pre-CI cortical activity and the amount of post-CI tinnitus improvement revealed that increased pre-CI connectivity between the AC and the OFC is a predictor of poor response to the improvement of tinnitus-related distress (Song et al., 2013b). In particular, a previous qEEG study has demonstrated that the OFC were more activated in highly distressed tinnitus patients than in less distressed patients for the delta band (Song et al., 2015b). In this regard, decreased activity in the OFC for the delta band after CI may be associated with the improvement of the TQ score in our case series.

One possible bias that might be crucial in the interpretation of the comparison between the CI-on condition and pre-operative baseline is the baseline activity of the subjects. In other words, the differences detected in the analysis above might also be partly affected by the changes in the baseline activity in the subjects that might have not be detected in the analysis comparing the CI-off condition and pre-operative baseline. To further clarify this issue, future studies comparing these conditions repeatedly in a larger number of subjects or comparing the CI-off condition and pre-operative baseline serially at different time points after turning the device off should be performed.

### “Dynamic” Cortical Activity Modulation by Peripheral Reafferentation

Although CIs starkly improved tinnitus and distress in our current patients with SSD, when they were turned off, the NRS tinnitus loudness and the TQ score returned to levels close to those measured pre-operatively. Moreover, the CI-on condition resulted in significantly decreased activity in the right A1 and A2 and the right OFC for the delta band and in the right A1 and A2, left A2, left TPC, left IFC, and left OFC for the gamma band, compared with the CI-off condition. In addition to the role of A1/A2 in tinnitus perception and of the OFC in tinnitus-related distress described above, the left TPC and IFC were significantly deactivated when the CI was on. The TPC contributes to the processing of auditory concepts (Bonner and Price, 2013) and increased pre-operative activity of the TPC was found to be a negative predictor of tinnitus loudness improvement in SSD patients after CI (Song et al., 2013b). The aggravation of tinnitus loudness after turning off the CI device may be partly due to reactivation of the left TPC. Meanwhile, the left IFC is involved in non-spatial auditory cognition and congruity (Michelon et al., 2003) or cognitive reappraisal (Wager et al., 2008). In a previous meta-analysis of PET studies in tinnitus, the IFC, or the ventrolateral prefrontal cortex, has been found to be commonly activated in tinnitus patients (Song et al., 2012). Therefore, cognitive processing of tinnitus may have been disinhibited in the current subjects after turning off the CI device, and this disinhibition may have manifested as the aggravation of tinnitus loudness.

When the pre-operative baseline and post-operative CI-off conditions were compared, neither subjectively perceived tinnitus loudness/distress nor source-localized activity showed statistically significant differences. CI-induced peripheral reafferentation was effective in alleviating tinnitus only when the device was actively functioning, at least until 6 months post-operatively. In other words, CI-related improvements in tinnitus may be associated with peripheral auditory reafferentation-induced dynamic suppression of tinnitus-related maladaptive cortical activity.

### Limitations of the Current Study and Proposed Future Studies

To our knowledge, this is the first study comparing pre- and post-operative cortical activity and functional connectivity in SSD patients who underwent CI. Although we found several significant findings, there are several limitations that should be further investigated in future studies. First, only four patients were included in this study. Although we found several cortical areas that showed significantly decreased activity and functional connectivity for the post-operative CI-on condition, as compared to the pre-operative baseline or post-operative CI-off conditions, we may have failed to discover other crucial areas that also contribute to the improvement of tinnitus, due to the limited statistical power. Additionally, the lack of differences between CI-off and baseline conditions or no significant correlations between cortical activity changes and percent improvement in tinnitus loudness or TQ score might have been due to small subject number-related insufficient statistical power inherent in the current study. Second, the current study revealed dynamic tinnitus suppression by peripheral sensory reafferentation, and these results should be reevaluated in a future study with both a larger number of subjects and a longer follow-up period. In the current study, the subjects’ post-operative EEGs were measured 6 months post-operatively, which may not have been long enough to observe possible central changes induced by continuous peripheral stimulus. Further studies in SSD subjects with CI, with follow-up periods of at least 12–24 months, should be performed to explore possible plastic changes. Third, all four subjects in the current study were coincidentally left SSD subjects. This may have affected the results because previous researchers reported that cortical activity differences from normal hearing peers are reported to be larger when the hearing loss occurred in the left ear compared with the right ear (Ponton et al., 2001; Hanss et al., 2009), and left and right unilateral sensorineural hearing loss subjects show different cortical activation patterns to sound stimuli (Schmithorst et al., 2005). Further studies comparing left- and right-SSD subjects with tinnitus after CI should be performed to further explore possible differences. Fourth, the lack of control group, composed of SSD subjects without tinnitus who underwent CI, limits the value of comparison between the CI-on and CI-off conditions. Future studies enrolling SSD subjects without tinnitus who underwent CI as a control group should be performed to further compare CI-on and CI-off conditions. Fifth, BL-ICA-based cleaning of the CI-on condition might have resulted in power decrease in the cleaned bands, and thus the direct comparison between the CI-on and CI-off conditions has inherent limitations. Future studies using auditory stimulation of the non-deaf side may give information on what extent the BL-ICA itself has an adverse effect on the interpretation of the results, and thus give us more precise results. Also, future studies using a similar study paradigm to the current study while measuring cortical activity changes by PET may give us additional precise information, as PET is not affected by device-related artifacts.

## Conclusion

Taken together, our data demonstrated that the CI-on condition resulted in decreased cortical activity compared with both the CI-off and pre-operative baseline conditions, particularly in areas such as the A1/A2 and the OFC. Also, decreased functional connectivity between the A1/A2 and the PCC were observed in the CI-on condition compared with pre-operative baseline. However, the CI-off and pre-operative baseline conditions showed no significant differences with regard to source-localized activity and functional connectivity. In this regard, CI may alleviate tinnitus in patients with SSD not by sound stimuli-induced cortical plastic changes, but by suppressing abnormally active tinnitus-related cortical regions by dynamic peripheral reafferentation.

## Author Contributions

J-JS led the analysis and interpretation of the results, and drafted the first manuscript. K-JP, HC, and J-WC conceived the investigation, revised the manuscript for important intellectual content. KK, WS, S-YL, GM, PVdH, DDR, and SV contributed to all aspects of the investigation, including methodological design, data collection and analysis, interpretation of the results, and revision of the manuscript for important intellectual content. All authors approved the final version of the manuscript and agree to be accountable for all aspects of the work.

## Conflict of Interest Statement

The authors declare that the research was conducted in the absence of any commercial or financial relationships that could be construed as a potential conflict of interest. The reviewer RP and handling Editor declared their shared affiliation, and the handling Editor states that the process nevertheless met the standards of a fair and objective review.

## Footnotes

1 http://www.uzh.ch/keyinst/NewLORETA/Software/Software.htm

2 http://www.nitrc.org/projects/bnv/
