# Corrigendum: A Quantitative Electroencephalography Study on Cochlear Implant-Induced Cortical Changes in Single-Sided Deafness with Tinnitus

**DOI:** 10.3389/fnhum.2018.00046

**Published:** 2018-02-20

**Authors:** Jae-Jin Song, Kyungsoo Kim, Woongsang Sunwoo, Griet Mertens, Paul Van de Heyning, Dirk De Ridder, Sven Vanneste, Sang-Youp Lee, Kyung-Joon Park, Hongsoo Choi, Ji-Woong Choi

**Affiliations:** ^1^Department of Otorhinolaryngology-Head and Neck Surgery, Seoul National University Bundang Hospital, Seongnam, South Korea; ^2^Department of Information and Communication Engineering, Daegu Gyeongbuk Institute of Science and Technology, Daegu, South Korea; ^3^Department of Otorhinolaryngology and Head and Neck Surgery, University Hospital Antwerp, Edegem, Belgium; ^4^Department of Surgical Sciences, Section of Neurosurgery, Dunedin School of Medicine, University of Otago, Dunedin, New Zealand; ^5^Lab for Clinical and Integrative Neuroscience, School of Behavioral and Brain Sciences, The University of Texas at Dallas, Richardson, TX, United States; ^6^Department of Robotics Engineering, Daegu Gyeongbuk Institute of Science and Technology, Daegu, South Korea

**Keywords:** single side deafness, tinnitus, cochlear implantation, electroencephalography, dynamic peripheral reafferentation

In the published article, there was an error in affiliation 1. Instead of “Department of Otorhinolaryngology-Head and Neck Surgery, Seoul National University Hospital, Seoul, South Korea”, it should be “Department of Otorhinolaryngology-Head and Neck Surgery, Seoul National University Bundang Hospital, Seongnam, South Korea”. The authors apologize for this error and state that this does not change the scientific conclusions of the article in any way.

The original article has been updated.

## Conflict of interest statement

The authors declare that the research was conducted in the absence of any commercial or financial relationships that could be construed as a potential conflict of interest.

